# Supplementary material for: Bacterial–fungal interactions in the neonatal gut influence asthma outcomes later in life
Source: eLife. 2021 Apr 20;10:e67740. doi: 10.7554/eLife.67740 (PMC8075585; doi:10.7554/eLife.67740)
Supplement: Transparent reporting form [file elife-67740-transrepform.docx]

***eLife’s* transparent reporting form**

We encourage authors to provide detailed information *within their submission* to facilitate the interpretation and replication of experiments. Authors can upload supporting documentation to indicate the use of appropriate reporting guidelines for health-related research (see [EQUATOR Network](about:blank)), life science research (see the [BioSharing Information Resource](about:blank)), or the [ARRIVE guidelines](about:blank) for reporting work involving animal research. Where applicable, authors should refer to any relevant reporting standards documents in this form.

If you have any questions, please consult our Journal Policies and/or contact us: [editorial@elifesciences.org](about:blank).

**Sample-size estimation**

- You should state whether an appropriate sample size was computed when the study was being designed
- You should state the statistical method of sample size computation and any required assumptions
- If no explicit power analysis was used, you should describe how you decided what sample (replicate) size (number) to use

Please outline where this information can be found within the submission (e.g., sections or figure legends), or explain why this information doesn’t apply to your submission:

No explicit power analyses were used in the manuscript. CHILD Study samples were selected based on availability. Animal experiments were repeated at least twice (usually three times) to ensure reproducibility of results and to increase sample sizes. Given that the experiments relied on animal breeding, it was not possible to predict the number of animals per experimental condition that we would obtain. However, we aimed to include at least two dams per treatment condition in each experiment.

**Replicates**

- You should report how often each experiment was performed
- You should include a definition of biological versus technical replication
- The data obtained should be provided and sufficient information should be provided to indicate the number of independent biological and/or technical replicates
- If you encountered any outliers, you should describe how these were handled
- Criteria for exclusion/inclusion of data should be clearly stated
- High-throughput sequence data should be uploaded before submission, with a private link for reviewers provided (these are available from both GEO and ArrayExpress)

Please outline where this information can be found within the submission (e.g., sections or figure legends), or explain why this information doesn’t apply to your submission:

This information can be found in the figure legends and additional information is provided in the Methods section of the submission. No outliers were removed and all data was included when measurement of the parameter in question was performed. Sequencing data will be made publicly available upon publication and is deposited in the NCBI Sequence Read Archive database.

**Statistical reporting**

- Statistical analysis methods should be described and justified
- Raw data should be presented in figures whenever informative to do so (typically when N per group is less than 10)
- For each experiment, you should identify the statistical tests used, exact values of N, definitions of center, methods of multiple test correction, and dispersion and precision measures (e.g., mean, median, SD, SEM, confidence intervals; and, for the major substantive results, a measure of effect size (e.g., Pearson's r, Cohen's d)
- Report exact p-values wherever possible alongside the summary statistics and 95% confidence intervals. These should be reported for all key questions and not only when the p-value is less than 0.05.

Please outline where this information can be found within the submission (e.g., sections or figure legends), or explain why this information doesn’t apply to your submission:

Information on statistical tests is included in the Methods section and Figure legends. The results of statistical tests are reported in the Figures and Figure legends. Source data for all figures has been provided in the submission.

(For large datasets, or papers with a very large number of statistical tests, you may upload a single table file with tests, Ns, etc., with reference to sections in the manuscript.)

**Group allocation**

- Indicate how samples were allocated into experimental groups (in the case of clinical studies, please specify allocation to treatment method); if randomization was used, please also state if restricted randomization was applied
- Indicate if masking was used during group allocation, data collection and/or data analysis

Please outline where this information can be found within the submission (e.g., sections or figure legends), or explain why this information doesn’t apply to your submission:

Information relevant to this point is included in the Methods section of the submission. Histological pathology was scored by a blinded investigator. Animals (dams and their pups) were assigned as “Control” or “Pichia” animals randomly at birth.

**Additional data files (“source data”)**

- We encourage you to upload relevant additional data files, such as numerical data that are represented as a graph in a figure, or as a summary table
- Where provided, these should be in the most useful format, and they can be uploaded as “Source data” files linked to a main figure or table
- Include model definition files including the full list of parameters used
- Include code used for data analysis (e.g., R, MatLab)
- Avoid stating that data files are “available upon request”

Please indicate the figures or tables for which source data files have been provided:

Source data is provided for all figures in the manuscript. 16S sequencing data has been deposited in the SRA database and will be available upon publication. Reviewers can access the sequencing data here: [http://www.ncbi.nlm.nih.gov/bioproject/624902](about:blank).

RNA-eq data has been deposited under Bioproject ID PRJNA706731.
